# Swarm Intelligence Integrated Graph-Cut for Liver Segmentation from 3D-CT Volumes

**DOI:** 10.1155/2015/823541

**Published:** 2015-11-24

**Authors:** Maya Eapen, Reeba Korah, G. Geetha

**Affiliations:** ^1^Department of Computer Science and Engineering, Jerusalem College of Engineering, Chennai 600100, India; ^2^Department of Electronics and Communication Engineering, Alliance University, Bangalore 562106, India

## Abstract

The segmentation of organs in CT volumes is a prerequisite for diagnosis and treatment planning. In this paper, we focus on liver segmentation from contrast-enhanced abdominal CT volumes, a challenging task due to intensity overlapping, blurred edges, large variability in liver shape, and complex background with cluttered features. The algorithm integrates multidiscriminative cues (i.e., prior domain information, intensity model, and regional characteristics of liver in a graph-cut image segmentation framework). The paper proposes a swarm intelligence inspired edge-adaptive weight function for regulating the energy minimization of the traditional graph-cut model. The model is validated both qualitatively (by clinicians and radiologists) and quantitatively on publically available computed tomography (CT) datasets (MICCAI 2007 liver segmentation challenge, 3D-IRCAD). Quantitative evaluation of segmentation results is performed using liver volume calculations and a mean score of 80.8% and 82.5% on MICCAI and IRCAD dataset, respectively, is obtained. The experimental result illustrates the efficiency and effectiveness of the proposed method.

## 1. Introduction

In recent years, liver ailments have been diagnosed as one of the common internal malignancies. The typical clinical treatments for such ailments are living donor liver transplantation (LDLT), oncological liver sectioning, and chemotherapy. All these surgical treatments are quite intricate and vulnerable to life threatening complications. While all these procedures differ in procedural implementation, they all rely heavily on precise identification and segmentation of liver regions from contrast-enhanced 3D computed tomography (CTA) volumes, which provides detailed anatomical cues with a series of 2D image slices. Accurate segmentation of liver from surrounding tissues and organs on these 2D slices is necessary for measurement of [1] liver volume and vasculature analysis, which decides further treatment directions.

In clinical practices, the acquired 2D images are interpreted by radiologists, who demarcate the organ boundaries on each CT slice either manually or interactively to accomplish segmentation. Manual segmentation is time consuming, tedious, and operator intensive. The accuracy of segmentation results highly relies upon the operators' experience and expertise. Recent advances in computerized CT image segmentation improve the segmentation efficacy manyfold offering several advantages over manual segmentation. In spite of its overwhelming merits over manual segmentation, automatic segmentation has to incorporate complex algorithms and techniques to offer high level of accuracy [1, 2] and to overcome the following challenges imposed by abdominal CT images. (1) Liver tissue is surrounded by adhesive soft tissues like gall bladder, right kidney, heart, stomach, and abdominal wall with similar intensity and sharing ambiguous boundaries with the liver. (2) Liver suffers from intensity diversity problem due to the presence of complex vasculature and liver tissue. (3) The pathological situations of the organs surrounding the liver can result in different contrast on an array of slices even in the same dataset. For the clinical end users, the aspects such as segmentation accuracy, minimal user interaction, and capability to process all potential cases are essential. Furthermore, a slice-by-slice segmentation procedure is time consuming and the segmentation results on each slice are independent of each other. Therefore, 3D segmentation is required for more efficient and accurate result [3].

Any methods proposed should incorporate a well-designed algorithm with intuitive and consistent interactive software for the clinical community. Based on implementation methodology, the available automated segmentation methods can be classified [2] into (1) low-level information based methods and (2) prior knowledge guided methods. Low-level information, such as gradient, texture, and intensity based methods [4–6], employs heavily pre- and postprocessing steps to overcome blurred boundary and boundary leakage conditions inherent to the CT images. Very often they default to unsuccessful semiautomatic operator assisted interactive demarcation of boundaries. Prior knowledge guided methods on the other hand employ super imposing techniques of imposing the statistical shapes and templates [7–9] acquired or derived priorly over the CT image artifacts to demarcate the boundaries. While this method offers significant advantage over the low-level information method, it has several limitations due to the inherent high variability of intra- and interpatient liver shape, for example, resected livers. In spite of all of its dynamic flexibility and highly versatile search algorithm with large array of liver shape models, this method does not offer high rate of success.

Given these problems and requirements, in recent years, the popular methods in energy minimization or variational framework [3] such as level set based methods and graph based methods are widely adopted. From liver segmentation perspective, traditional level set methods usually produce local minima convergence of the energy function and are sensitive to contour initialization. Meanwhile, the neighboring soft tissue of the liver may need to be segmented to avoid boundary leakage [10]. In contrast, graph-cut model demonstrates a suitable framework and powerful tool to produce globally optimal binary segmentation with the merits of global optimization and practical efficiency [11]. The choice of weighting parameter for the data and smoothness terms in the energy function of the graph-cut impacts the model's segmentation performance. In [3], voxel intensity and path distance are incorporated in the data term to accomplish liver segmentation. In [12], region constrained energy terms are imposed in order to improve the weighting parameter selection strategy for the energy function to achieve segmentation of cerebral white matter. Recently, [13] suggested the usage of derivative of Laplacian of Gaussian weighting function (DroLoG) as the weighting function in traditional graph based approaches. The approach demands contrast enhancement as a preprocessing stage for reliable segmentation. In [14], an approach is presented which uses the semantic information obtained from learned random forest (RF) to define a smoothness cost for graph-cut based prostrate segmentation. When it comes to liver segmentation, graph-cut models are incapable in the presence of seriously vague or blurred boundaries and similar intensities between liver and its surrounding organs. More importantly, the model performance is sensible to the energy function parameters (data and smoothness terms) that are derived from interactive or empirical information.

To extend the robustness of graph-cut for liver segmentation, this study endeavors to implement improvisation of the data and smoothness terms in the graph-cut energy formulation through domain knowledge and computational intelligence approaches. The domain knowledge on liver's location and spatial connectivity [3] are unified with local intensity model in the form of probabilistic similarity measure based data term. We now state another potential benefit of the proposed method. The weighting function that plays a major role in deciding the robustness of the boundary term becomes a major concern, if Gaussian function is used for such purpose [13]. In the proposed work, the precision of the boundary term is improved in low-contrast images with an image enhancement stage. Generally, the traditional methods first apply the filtering process which leads to degradation in image quality such as blurring and smoothing of edges. We adopt a different approach. Unlike filtering, we utilize region appearance profile in a constructive manner for enhancement. Enhancement operation is performed relative to the region statistics of ROI (user-defined object region) and local contextual information present in the individual 2D slices of the input sequence [2]. The boundary knowledge of the enhanced image is then enumerated using computational intelligent model such as swarm intelligence. As a whole, our main contribution is the proposition of an improved graph-cut algorithm with domain adaptive data and boundary term parameters.

The paper is organized as follows. An overview of 3D graph-cut segmentation framework as described in [11] is given in Section 2.  Section 3 describes materials and methods, which includes the initialization of graph-cut segmentation algorithm, construction of domain knowledge adaptive data term, and the ROI-based image enhancement for computing boundary term. In Section 4, the experimental results along with performance evaluation of the proposed approach are reported and discussed.  Section 5 presents concluding remarks.

## 2. 3D Graph-Cut Segmentation

Let the data volume *V* be considered as an undirected graph, *𝒢* = 〈*𝕍*, *𝔼*〉, where *𝕍* = {*𝒫*, *𝒮*, *𝒯*} is defined as the set of nodes or vertices and *𝔼* is the set of edges. The vertices are the graph's representation of the image voxels and the edges are the representation of each voxel's relationship with its neighboring voxels. Each pair of nodes (*𝓊*, *𝓋*) ∈ *𝔼* in a neighborhood *𝒩* is connected by a link named *𝓃*-link which is assigned with a nonnegative weight term *𝒦*(*𝓊*, *𝓋*) that is designed to promote the spatial coherence in the neighborhood *𝒩* of voxels. *𝒦*(*𝓊*, *𝓋*) is typically defined as

(1) $$\begin{array}{c} {K\left(u,v\right)}_{}=e^{{-\left(I_{u}-I_{v}\right)}^{2}/2\sigma^{2}}\;\left(v\in N_{u\left(6\right)}\right), \end{array}$$

where *I*_*u*_ and *I*_*𝓋*_ are the intensity values of voxels *u* and *𝓋* and *𝒩*_*𝓊*_ is the 6-connected neighborhood of *𝓊* in 3D space.

Consider two specially designated nodes, referred to as terminal nodes; the source node *𝒮* represents the foreground region *𝒪* (in our case liver) and the sink node *𝒯* represents the background region *ℬ*. Each node *𝓊* ∈ *𝒫* is connected to the terminal nodes by two links called *𝓉*-links and is weighted by data or region term *𝒟*_*u*_, which is interpreted as degree of fitness with the foreground and background regions. It is defined by

(2) $$\begin{array}{c} D_{u}\left(\;\omega \;\right)=-\log Pr\left(\;x_{u}\;|\;\omega \;\right), \end{array}$$

where Pr⁡(*x*_*u*_∣**ω**) denotes the likelihood estimate of observed data feature *x*_*u*_, given that voxel *u* belongs to class **ω**.

A cut *𝒞* in *𝒢* involves cutting *𝓃*-links and *𝓉*-links to contribute a label *𝒪* or *ℬ* to each voxel *u* in the image and hence results in the segmentation of volume *V*. The energy of the cut *𝒞* is defined as

(3) EnfC=∑u∈VDuωu+λ∑u,v∈NKu,vδωu≠ωv,

where *δ* holds value 1 if the condition inside the parenthesis is true and 0 otherwise. Optimal 3D segmentation is achieved by searching for a cut with minimal energy. The min-cut can be achieved by max-flow algorithm in polynomial time [15, 16].

## 3. Material and Methods

### 3.1. Anatomical Knowledge Based Initialization

**Figure 1:**
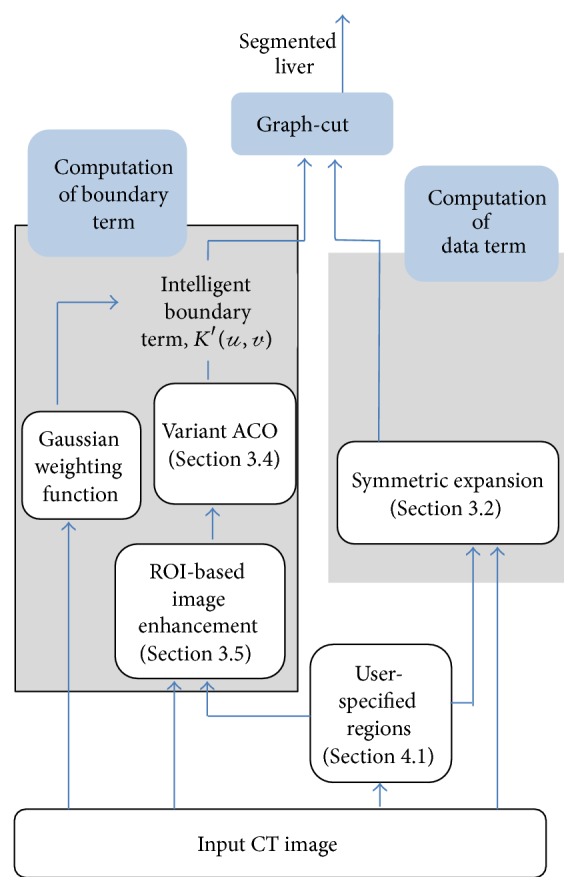
Overview of domain knowledge and swarm intelligence (DKSI) integrated graph-cut.

Based on the notion of specific anatomical knowledge such as liver location, intensity distribution, and size [3], it is possible for the user to localize liver and background region in the abdominal CT image. To initialize the proposed domain knowledge and swarm intelligence (DKSI) integrated graph-cut method depicted in Figure 1, the user is required to select multiple seed regions on ROI (liver) and background tissues (Figure 5). Further discussion with data preparation and initialization is given in Section 4.1.

### 3.2. Computation of Domain Knowledge Centric Data Term

The image data term *𝒟*_*u*_ in the energy function (see (3)) is the individual penalty in labeling a voxel as *𝒪* or *ℬ*. It is computed with the probability density function defined on the basis of prior knowledge. When the intrinsic intensity feature alone cannot separate tissues, a global histogram analysis can often provide diversifying information [1]. To maximally support the exclusion of complex background regions and to distinguish liver region from the overlapping intensity range, we use intensity range weight criteria as the first piece of prior anatomical or domain knowledge. Owing to the physics of CT imaging, the intensities of the abdominal organs such as liver correspond to {*ς*, *ξ*} interval [2]. Even though it is difficult to fix sharp bounds for this interval [2], the rough bounds are obvious and intensities outside the interval explicitly indicate background tissue. In this view, an intensity based weight function is written as [2]

(4) Iw=10<Iw~<θeqIw~otherwise,Iw~=I−ςξ−Iξ−ς,

in which *q* is a constant and *θ* regulates the breadth of dubitable area in the intensity interval [2]. With offline training, set *θ* = (1/7) × (6/7) in order to put forth high confidence level on the central 3/4 of the intensity range. Nevertheless, due to inherent intensity overlapping of liver with the neighboring organs, the intensity interval will inevitably include nonliver tissues. Therefore, extra care should be given to regions in close proximity to liver intensity interval margins.

A second piece of domain knowledge is that “the liver is spatially continuous in the abdominal cavity.” This continuity criterion can be approximated by the spatial connectivity property defined on intensity and Euclidean distance feature [3]. The connectivity metric is deduced relative to the user-specified liver region and background region. Specifically, if a point is spatially related to a user-specified region, most probably they both belong to the same region; that is, it possesses high membership degree under the region label.

The proposed similarity measure, Pr⁡(*x*_*u*_∣*ω*), combines the path based connectivity features [3] with intensity based weight function (see (4)) to bring out accuracy in the model. The similarity score between a voxel and user-specified seed regions is iteratively calculated through symmetric expansion along a 6-connected neighborhood from the specified seed regions. Let *Q*_liverseed_^1^ denote the set of liver seeds and *Q*_*l*_^1^ is the set of unvisited neighborhood sets of *Q*_*l*−1_^1^ at the *l*th expansion; then, ∀*l* ∈ *Q*_*l*_^1^, the likelihood function can be written as in [3]

(5) Pr⁡xu ∣ ω=O=maxl−1∈Ql−11⁡exp−Iwμ1Intl−Int−l−12/disl,l−1+Disl−1Q01=Qliverseed1,

where Int_*l*_ represents intensity of voxel and dis_*l*,*l*−1_ measures the Euclidean distance between *l* and *l* − 1 in its 6-connected neighborhood. The intensity value ${\overset{-}{Int}}_{l}$ is updated with the average intensity along the path from *l* − 1 to the liver seeds *Q*_liverseed_ and refines the aggregate path distance Dis_*l*_ with the Euclidean distance dis(*l*, *l* − 1) as

(6) Int−l=Intl+Int−l−12,Disl=disl,l−1+Disl−1,

with initial condition ${\overset{-}{Int}}_{l}={Int}_{0}$. With *μ*_2_ and ∀*l* ∈ *Q*_*l*_^0^, the probability distribution Pr⁡(*x*_*u*_∣*ω* = *ℬ*) through expansion from the background seeds *Q*_non-liverseed_^0^ is computed. The approach avoids the repeated traversals through the entire image domain and thus has satisfactory processing speed. Moreover, the probability distributions are significantly influenced by the nearby user-specified seed regions to provide reliable solutions for intensity diversity and intra/interliver shape variability problem.

Except in the dubitable range, the intensity weight *I*_*w*_ boosts Pr⁡(*x*_*u*_∣*ω*) → *𝒪* on highly confident liver regions irrespective of false edges and thereby helps to jump over the local minima caused by false edges in the liver region. The Euclidean distance reduces the effects of global minima.

### 3.3. Computation of Boundary Term

**Figure 2:**
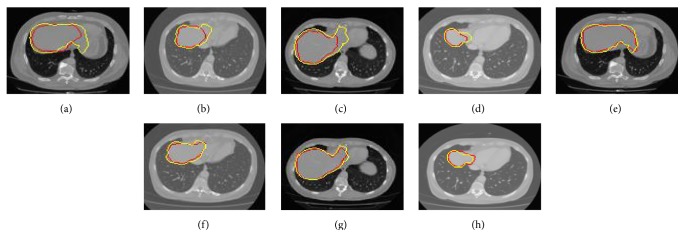
Segmentation results on “special slices.” Ground truth in yellow color and segmentation results in red color. (a)–(d) Segmentation with DKC (domain knowledge centric) graph-cut and (e)–(h) results of DKSI (domain knowledge and swarm intelligence) integrated graph-cut.

In graph-cut, a weighting function is used to map changes in image intensities to the edge weights or the boundary term. The weights of the edges measure the similarity between two connected vertices. That is, the boundary term *K*(*𝓊*, *𝓋*) is interpreted as the penalty for the discontinuity between neighboring pixels *𝓊* and *𝓋*, the correctness of which is typically required to guarantee segmentation results with smooth boundaries [11]. In other words, the success of the graph-cut algorithm greatly depends on the variation in the capacities or edge weights in the graph, which in turn depends on the existing contrast in the image. When *𝓊* and *v* become more similar to each other, the cost of penalty is larger. The penalty cost decreases and approaches zero, when the voxels *𝓊* and *𝓋* are very dissimilar. What is more, the penalty term is sensitive to pixel intensity similarity, which can be measured based on gradient direction, Laplacian zero-crossing, gradient direction, and so on. Though Gaussian weighting function (see (1)) is a good tool for most cases in liver segmentation, it produces undesirable segmentation results along the blurred or confusing boundaries if used alone. More importantly, the performance of any graph-cut based segmentation depends on the choice of this weighting function for the correct identification of inexpensive edges [13]. Under certain pathological conditions, the minimization of domain knowledge centric (DKC) data term and Gaussian weighted boundary term based energy function results in imprecise segmentation results. The presence of blood inside the heart and liver leads to similar intensity values. The boundary can be hardly distinguished with the human eye. On such slices, referred to as “special slices,” the Gaussian based weighting function results in imprecise boundary weights which in turn results in cuts through a uniform region and thus underestimates the volume (Figures 2(a)–2(d)).

In this case, the anatomical and pathological situation of the patient is so important that the employed domain knowledge constraints alone cannot well guide the demarcation of liver from its adjacent tissues. Therefore, a computational intelligent model such as ant colony optimization (ACO) is adapted to detect and optimize genuine boundary edges in the image.

In recent years, estimating the adaptive regularization weights from the observed images has become vital to obtain optimal performance [17]. As mentioned earlier, a decreased magnitude of the boundary term indicates that the corresponding edge is more likely to be segmented. A new quantization function, *B*_*u*,*v*_, based on swarm intelligence of a variant ant colony optimization (ACO) algorithm is proposed here to optimize the Gaussian boundary term and is defined as

(7) $$\begin{array}{c} B_{u,v}=\left(\;1-\tau_{u,v}\;\right). \end{array}$$

We found that the attractiveness matrix, *τ* (∈[0.001,0.999]), derived with swarm intelligence variant ACO (Section 3.4) as a quantitative index, is particularly useful to adaptively regularize the graph-cut energy minimization. The left branch of Figure 1 illustrates the integration of swarm intelligence with the Gaussian boundary term. We denote by *K*′(*𝓊*, *𝓋*) = *B*_*u*,*v*_ *K*(*𝓊*, *𝓋*) the modified boundary term, in which *B*_*u*,*v*_ is the swarm intelligence factor. It determines the suitability of a weighting function to a particular edge for its detection. Moreover, *τ*_*u*,*v*_ depicts the confidence rate of edge (*𝓊*, *𝓋*) ∈ *𝔼* being part of the optimized solution. To be specific, the smoothness term is modulated by swarm intelligence. The contribution of the weight function, *B*_*u*,*v*_, to the edge capacity gradually decreases for edges away from the true boundary. On the one hand, high *τ* along the boundaries decreases the magnitude of the boundary term to ensure smooth cut along the genuine boundaries. Also, the domain knowledge and intensity model based data term suppresses the negative effects of the background edges and false edges in the liver region. Then, the energy function in (3) is transformed into

(8) EnfC=∑u∈VDuωu+λ∑u,v∈NBu,vKu,vδωu≠ωv.

**Figure 3:**
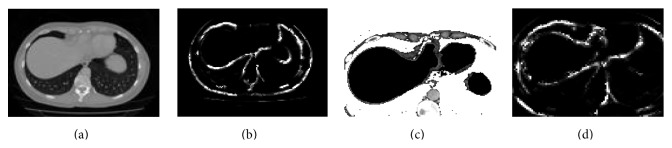
Edge-detection results. (a) Input: sample special slice. (b) Output of normal ACO. (c) Input: ROI-based image of (a). (d) Output of variant ACO.

Figure 3 shows the results of variant ACO based boundary detection on a “special slice.” Figures 2(e)–2(h) present the segmentation results on “special slices” using domain knowledge and swarm intelligence (DKSI) integrated graph-cut.

### 3.4. Boundary Detection and Optimization Using ACO

Computational intelligent systems (CIS) play a crucial role in the field of medicine. CIS like neural networks, machine learning, swarm intelligence, genetic algorithms, and fuzzy logic are applied to different medical problems and in various domains like neurology, gynecology, cardiology, oncology, and ophthalmology. The CIS are widely applied for segmentation and classification of medical images [18–21]. Significantly visible research has been performed over applying ACO for the segmentation of objects from images. A remarkable milestone in computerized segmentation of heart and left ventricle is reported by [19]. This work integrates the ACO with active contour model to reach the best feasible boundary with minimum energy value. Sahoo and Chandra [19] also introduced the applicability of ACO on graph based systems. In this context, this paper performs experiments with the ACO approach for liver boundary detection combined with the energy minimization process of graph-cut to obtain remarkable performance improvements.

The basic idea behind ACO is movement of ants. All the ants follow the same path with the help of trails called pheromone left by the predecessor ants. The succeeding ants utilize this pheromone to find its path. Each ant incrementally constructs a solution for the problem.

In ACO based edge detection [20], the movement of the ants is driven by heuristic information to establish a pheromone matrix iteratively, which depicts the cost of the solution. From the results of normal ANT colony algorithm on raw “special slices,” we found that some improper edges are detected (Figure 3(b)).

To overcome this, a variant ACO algorithm can be used to detect the genuine edges of the image. Variant ACO is an alteration of normal ACO algorithm. Variant ACO algorithm works on the output of ROI-based enhanced image (Section 3.5).

**Lemma 1 (see [20]).**

Pheromone *τ* is finite; that is, *τ*_min_ ≤ *τ* ≤ *τ*_max_.

Following Lemma 1 avoids stagnation problem. The finite interval according to Max-MinAS algorithm [21] is set as [0.001,0.999]. The detailed pseudocode for the proposed variant ACO is given in Algorithm 1. Usually, the information in image content prevails pheromone trail. Therefore, *β* > *α* is a reliable choice, and in our experiments, we assume *α* = 1 and *β* = 2 [18]. Moreover, parameters *τ* ^(0)^, *φ*, and *ρ* are always small to allow consistent change of pheromone trails between the iterations. Our experiment adopts *τ* ^(0)^ = 0.000001, *φ* = 0.2, and *ρ* = 0.3 [18]. The core of the algorithm is the pheromone updating process, which is intended to provide a greater amount of pheromone for shorter routes. The decrease in the amount of pheromone on the edges during the local updating process of the normal ACO reduces the chance of some potential edges to be the part of final solution of ACO. The global updating process focuses on providing a greater amount of pheromone to shorter routes. In variant ACO, we follow a selective updating process, so that, for each local updating iteration, pheromone values of all edges remain unchanged and at each global updating operation (after a finite number of local iterations) pheromone levels for the edges belonging to the best-tour-so-far are increased, whereas the pheromone levels of edges that do not belong to the best-tour-so-far are decreased. By taking several alternative local paths, the algorithm chooses the global best path which gives the true boundaries of the image. The edges on the best path have high pheromone values as compared to the other path edges.  Figure 3(c) illustrates ROI-based enhanced image of the image in Figure 3(a). Figures 3(c) and 3(d) are the input and output of variant ACO.

### 3.5. ROI-Based Image Enhancement

To facilitate maximum discrimination of ambiguous edges, we establish the characteristics of each image voxel *u* = (*x*, *y*, *z*) ∈ *V* with the appearance of its small neighborhood, *𝒩*(*u*). To accomplish this, two discriminative region descriptors were chosen, namely, intensity, *I*(*u*), and Local Binary Pattern, LBP_6,1_^*ω*^. For CT images, intensity is recognized as the highly informative feature [2]. Since intensity alone cannot separate liver region from neighboring organs, a local neighborhood context can often provide better diversifying information. LBT is an excellent region discriminative feature for identifying spatial patterns and analyzing microstructures [22]. The paper [2] introduced parameter *ζ* to take into account the noisy feature of CT images, and it is written as

(9) $$\begin{array}{c} {LBP}_{M,R}^{\zeta }=\overset{M-1}{\underset{t=0}{\sum }}H\left(\;I_{t}-I_{c}-\zeta \;\right)2^{t}, \end{array}$$

where *I*_*c*_ represents grey level intensity value of the center voxel of a local neighborhood *I*_*t*_  (*t* = 0,1,…, *M* − 1), which represents the intensity values of *M* equally spaced voxels on a sphere of radius *R*, defining a spherically symmetrical local neighborhood set. However, [22] suggests using a circularly indexed neighborhood to guarantee rotationally invariant LBP descriptor. We empirically choose *ζ* = 1.6 in our experiments. Compared to voxel-wise features, patch-wise (texture) features like LBT can efficiently capture more discriminative local patterns. However, the patch-wise features are not enough to capture more distinct local context, thus making it difficult to distinguish multiple organ tissue voxels in a local neighborhood window. Hence, each voxel *u* ∈ *V* is characterized with distributions *C*_*u*_^*k*^  (*k* = 1,2) of the features *I*(*u*) and LBP_6,1_^*ζ*^ over a localized window defined around *u*, termed local region profile [2].

To further reduce the false positives, local region profile is combined with global region characteristic which is enumerated with the help of anatomical knowledge. Despite complex background cluster, medical organs possess relatively homogenous appearance properties. To encode this global characteristic, global appearance models *C*_*o*_^*k*^  (*k* = 1,2) with tolerable appearance variances *σ*_*o*_^*k*^  (*k* = 1,2) in the form of histogram criteria from the user-specified liver regions, that is, ROI *Q*_liverseed_^1^, or prior knowledge are defined [2]. *C*_*o*_^*k*^ and *σ*_*o*_^*k*^ are defined as mean appearance distribution and variance, respectively, over {*C*_*u*_^*k*^ : *u* ∈ *Q*_liverseed_^1^ ⊂ *Ω* (image  domain)}.

One of the efficient approaches to compute the similarity measure between the estimated global and local appearance probability distributions is Wasserstein distance (*W* ^1^) [23] based on measure theory. The result is two informative fields {*C* ^*k*^(*u*) : *u* ∈ *Ω*,  *k* = 1,2} named region characteristic classification certainty fields (RCFs) that are potential enough to impose region appearance constraints

(10) $$\begin{array}{c} C^{k}\left(\;u\;\right)=\frac{W^{1}\left(C_{u}^{k},C_{o}^{k}\right)}{\sigma_{o}^{k^{2}}}=\frac{\int_{M_{1}^{k}}^{M_{2}^{k}}\left|F^{k}\left(y\right)-G^{k}\left(y\right)\right|dy}{\sigma_{o}^{k^{2}}}, \end{array}$$

where *F* ^*k*^ and *G* ^*k*^ are the cumulative distribution functions of *C*_*u*_^*k*^ and *C*_*o*_^*k*^, respectively, and [*M*_1_^*k*^, *M*_2_^*k*^] denotes the value range of feature *k*.

Regarding the implementation details, an adaptive integral histogram data structure is utilized for the efficient computation of the classification certainty fields. Moreover, probability distribution deduced from histogram is definitely an easy approach [24] and is of great benefit for capturing local context information, reflecting good discretion of local mixture of multiple organs. Local neighborhood region with more nonliver structures results in high certainty field values. The certainty field controls the region context and enhances the weak edges by local contextual information. Figure 3(a) shows ROI-based image with *C*(*x*) = *C* ^1^(*x*) + *C* ^2^(*x*) [2], which shows clear identification of weak edges and high liver region homogeneity.

The certainty fields are potential enough to improve regional consistency and enhance weak edges with local contextual information. It is noteworthy that the method adjusts the effect of the regularization weight by adjusting the edge capacities individually.

## 4. Experimental Results and Discussion

### 4.1. Data Preparation and Initialization

The proposed method has been evaluated on several clinical abdominal CT volumes stored as DICOM images. The in-plane resolution of each volume is 512 × 512 pixels. The datasets are partly from 3D-IRCADb databases maintained by French research institute (http://www.ircad.fr/) and partly from MICCAI 2007 liver segmentation challenge. Owing to different protocols, the 3D datasets have various dimensionality; the inner slice spacing varies from 0.486 to 0.95 mm, the interslice distance varies from 1.0 to 3.0 mm, and the number of slices in the data volume varies from 20 to 352. The model was implemented on MATLAB 2013. The parameters are set as follows: *λ* = 0.15, *μ*_1_ = 12, *μ*_2_ = 8, and *σ* = 10.

**Figure 4:**
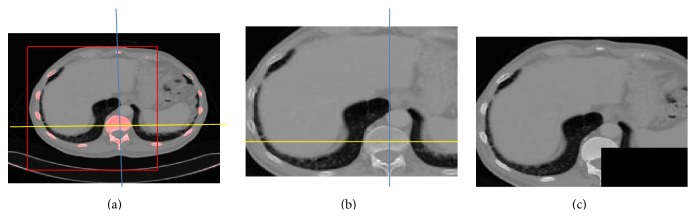
ROI extraction from 3D-CT data volume.

The ROI is defined within the original 3D volume using manually controlled minimum bounding box and crossline [3]. Initially, the original 3D volume is cropped against the red bounding box (Figure 4(a)) such that only the voxels inside the bounding box are conserved for the subsequent segmentation process. It maximally excludes the nonliver slices and voxels.

To further remove the unnecessary voxels from the computing space, a blue-yellow crossline is positioned at the centre of the spine with ribs identified as background region using thresholding operation. The direct elimination of the voxels located in the right-bottom quadrant of the crossline decreases more than three hundred thousand voxels from the computing space. Ultimately, the final ROI (Table 1) occupies only 31% of the original 3D volume and dramatically lowers the memory usage during segmentation.

**Figure 5:**
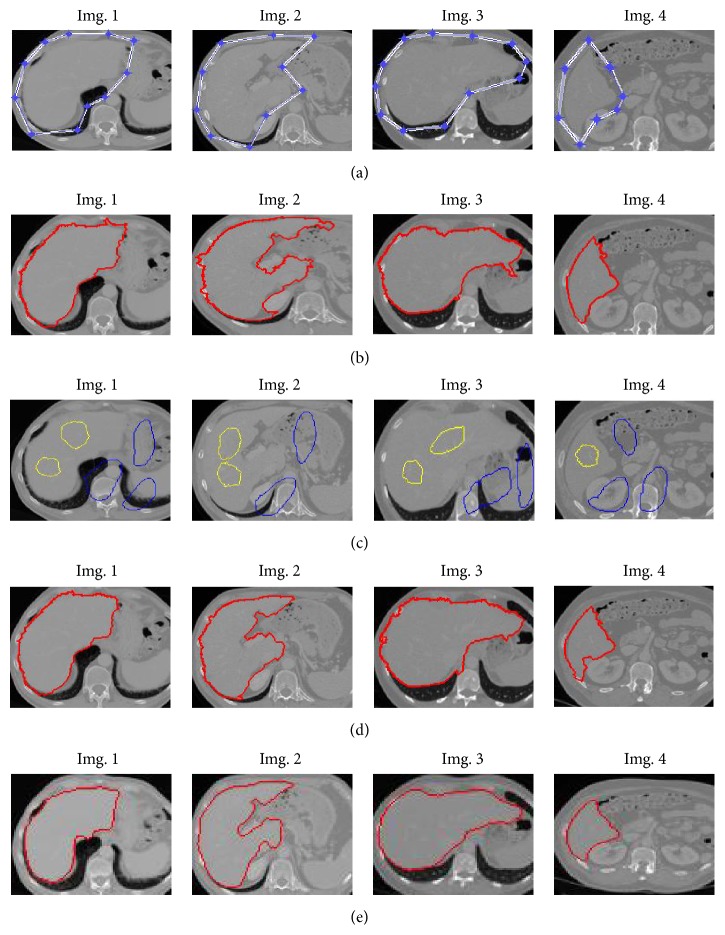
Comparison results. (a) Original 2D images with initial contours. (b) GCBAC. (c) Foreground initialization is in yellow and background is in blue. (d) GC. (e) The proposed method.

An easy-to-use initialization interface tool is designed to facilitate the incorporation of user domain knowledge. The user specifies multiple arbitrary shaped contours to denote foreground and background region. In Figure 5(c), region enclosed by yellow contours denotes the object seeds referred to as *𝒪*. Similarly, the background seeds *ℬ* result from regions inside the blue contours. In 3D volumes, the user can slide these contours to any other slice as ending slice [2]. From the starting slice to the ending slice, the specified initialization contours perceived a cylindrical shape.

To provide more effective and distinct context constraints, object regions are drawn around healthy liver tissues and vessels while the background contour is marked around highly ambiguous liver boundaries with neighboring organs. The regions are marked such that *ℬ*∩*𝒪* = *∅* and we model the labeling function *u* for the user-specified object (*𝒪*) and background region (*ℬ*) as

(11) $$\begin{array}{c} C\left(\;u\;\right)=\left\{\begin{array}{cc} 1, & \forall u\in O\\ 0, & \forall u\in B. \end{array}\right. \end{array}$$

### 4.2. Performance Comparison Results

In the first experiment, the proposed method is compared with similar energy minimization approaches for segmentation. Figure 5 presents the segmentation results on four typical slices from different CT volumes.

Figure 5(b) uses graph-cut based active contour approach (GCBAC, [25]) for segmentation, yet it achieved inaccurate results. In this approach, the active contour escapes over the local minima and provides more global segmentation results. Moreover, the approach is highly sensitive to contour initialization (Figure 5(a)) such that it should be placed strictly around the external boundary of the target object. Even with the ideal initialization, it produces undersegmentation and suffers high computation burden. Figure 5(c) is the original images with users' initialization for object and background regions for the methods in Figures 5(d) and 5(e). The segmentation results using globally optimal graph-cut (GC) [11] are illustrated in Figure 5(d). Besides blurred boundaries, GC model produces unsatisfactory results when target areas and neighboring tissues exhibit intensity similarity. The liver pixels near the kidney are wrongly labeled as background due to the high similarity to the background region and absence of obvious boundary.  Figure 5(e) exhibits the segmentation results obtained by our proposed method. In our framework, the simultaneous inclusion of ambiguous edge detection and domain information helps to demarcate the object and background region.

**Figure 6:**
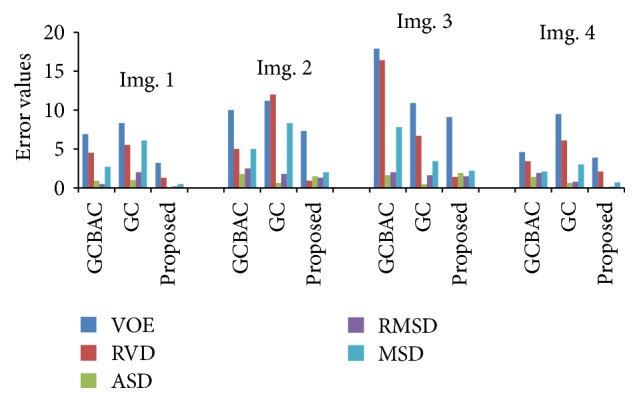
Performance comparison between three methods.

The third experiment deals with the quantitative evaluation of the segmentation results obtained using the proposed method. Since the application of the method demands the accuracy in liver volume calculation, quantitative evaluation utilizes five metrics including two volume error measures, VOE (Volume Overlap Error) and RVD (Relative Volume Difference), and three surface measures, ASD (Average Symmetric Surface Distance), RMSD (Root Mean Square Surface Distance), and MSD (Maximum Symmetric Surface Distance).  Figure 6 illustrates the performance comparison between GCBAC, GC, and the proposed method on the four typical slices in Figure 5 through five metrics. An ideal scoring result (i.e., zero for all five error measures) is worth 100 per metric, where the manual segmentation of average scoring results (6.4%, 4.7%; 1 mm, 1.8 mm, and 19 mm) is worth 75 per metric [26].

The results on 10 datasets of the MICCAI training volumes with reference segmentations available to the public are summarized in Table 2. It can be seen that the average errors for the five metrics are lower than that obtained using manual segmentation. The total score of the proposed method is 80.8 ± 3.38 and is also higher than state-of-the-art methods such as Kainmller et al. [8] (77 ± 9), Heimann and Meinzer [7] (67 ± 11), and Wimmer et al. [9] (76.38 ± 3.8), which are variants of energy minimization. The proposed method can perform efficient segmentation of arbitrary shaped liver regions.  Table 3 summarizes the quantitative results on the IRCAD database. In comparison to the low-level information based methods [4–6], no dependence on neighborhood tissue segmentation is foreseen in our method.

**Figure 7:**
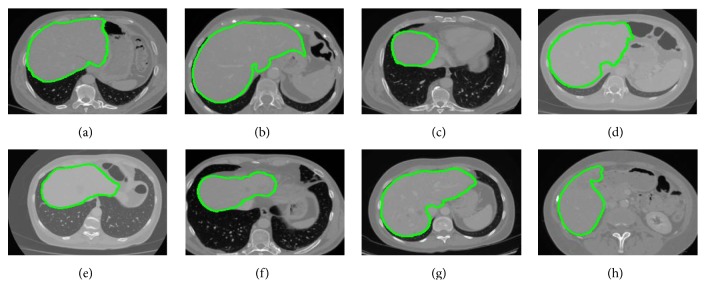
Segmentation results (marked in green) using the proposed method.

**Figure 8:**
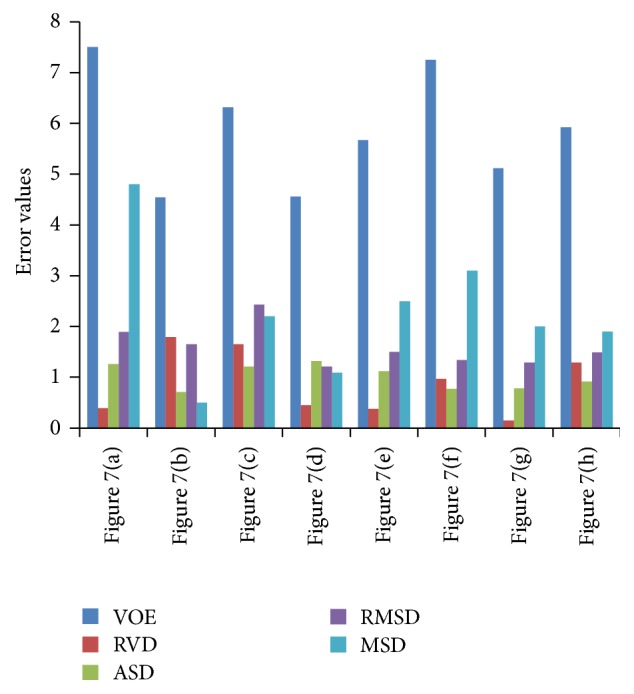
Quantitative evaluation results.

Figure 7 presents results of our segmentation and the corresponding quantitative valuation results are given in Figure 8. Volumetric visualization presents more information to the users for subsequent analysis treatment planning.

**Figure 9:**
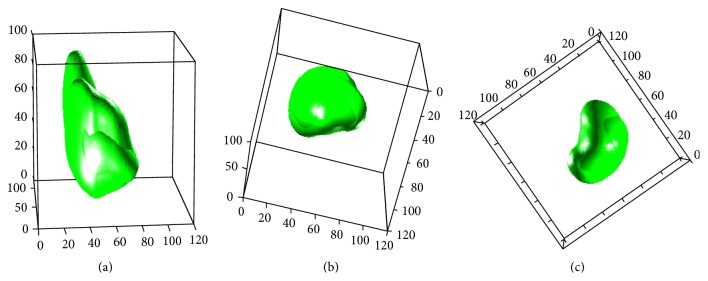
3D visualization of segmentation results: (a) axial view, (b) coronal view, and (c) sagital view.

An example of volumetric visualization of the segmented results using the proposed method is shown in Figure 9, which shows results in 3D, axial, coronal, and sagittal view.

### 4.3. Computational Complexity

The model also performed well when it came to efficiency. The runtime for each step of ten testing CT volumes is given in Table 1. The cropping operation results in subgraph by trimming a significant part of the 3D graph. The proposed algorithm is then applied on subgraph resulting in reduced execution time and better segmentation accuracy. Moreover, the proposed updating process of variant ACO reduces the execution time of normal ACO. The computer used for runtime measures had Intel Core 2 Quad CPU (2.66 GHz), 3.25 GB of RAM, and Windows 8.1 operating system.

### 4.4. Application to Spleen Segmentation

**Figure 10:**
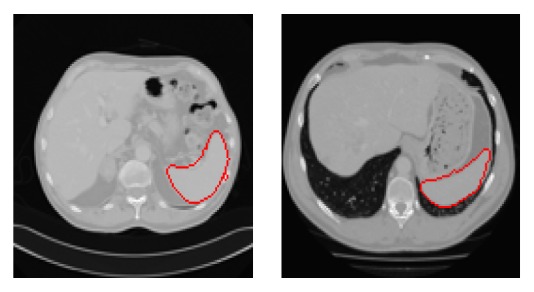
Segmentation results of spleen using the proposed method.

We further apply the proposed architecture for spleen segmentation as shown in Figure 10.

Even though there exist blurred boundaries and low contrast with the spleen, the proposed method can segment the spleen correctly.

## 5. Conclusion

In this paper, we have addressed the problem of liver segmentation from abdominal CTA images. From the medical point of view, segmentation of liver tissue is a prerequisite for diagnosis and treatment planning for liver ailments. We propose an improved graph-cut algorithm with the incorporation of information from both image cues and domain knowledge. Experimental results confirm the efficiency of the proposed method for segmentation of liver CT images from several challenging datasets and prove these advantages by comparing the new method with several energy minimization methods and state-of-the-art approaches. The contribution of the proposed method towards liver segmentation is limited when a large portion of liver boundary is fully missing. In future research, it is interesting to improve model performance with a probability learning model and spatial information. Moreover, the variant ACO algorithm will be accompanied with other social algorithms to improve the performance.

## Figures and Tables

**Algorithm 1:**
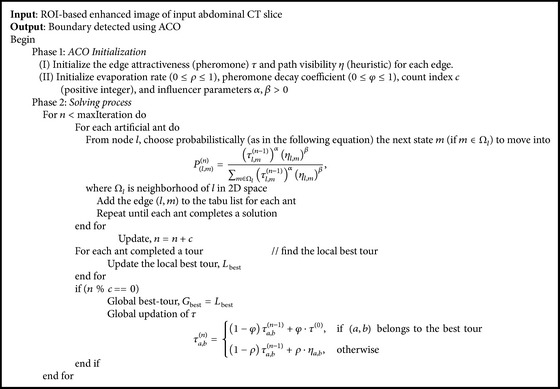
Variant ACO algorithm.

**Table 1:** Experimental results on efficiency.

Original 3D dataset	3D-ROI	Graph construction	Energy minimization
512 × 512 × 20	276 × 293 × 20	2 sec	9 sec
512 × 512 × 34	358 × 285 × 28	2 sec	12 sec
512 × 512 × 81	320 × 291 × 73	3 sec	15 sec
512 × 512 × 108	365 × 254 × 78	3 sec	17 sec
512 × 512 × 143	280 × 281 × 113	4 sec	21 sec
512 × 512 × 153	331 × 318 × 145	4 sec	34 sec
512 × 512 × 204	301 × 275 × 179	4 sec	23 sec
512 × 512 × 218	287 × 295 × 176	4 sec	21 sec
512 × 512 × 441	297 × 277 × 156	4 sec	37 sec
512 × 512 × 504	306 × 370 × 200	5 sec	40 sec

**Table 2:** Evaluation on the MICCAI dataset.

3D-CT dataset	VOE [%]	RVD [%]	ASD [mm]	RMSD [mm]	MSD [mm]	Score
1	8.28	0.43	1.33	1.98	16.8	76.5
2	5.43	1.32	0.95	1.69	17.52	80.3
3	6.47	1.74	1.08	2.43	22.75	74.96
4	6.73	0.88	0.42	0.95	15.14	85.08
5	5.8	0.34	0.57	1.09	13.16	85.77
6	7.15	0.54	0.96	1.65	17.1	79.96
7	5.12	0.45	0.76	1.28	13.35	84.65
8	6.28	0.9	0.92	1.49	17.84	80.7
9	4.16	3.53	0.72	1.26	16.7	81.5
10	5.25	0.73	0.93	2.23	19.8	79.07
Mean	5.54	0.93	0.78	1.47	15.6	80.8
Std. dev.	1.23	0.71	0.25	0.42	3.24	3.38

**Table 3:** Evaluation on IRCAD dataset.

3D-CT dataset	VOE [%]	RVD [%]	ASD [mm]	RMSD [mm]	MSD [mm]	Score
1	5.1	2.5	0.7	1.4	16.1	81.73
2	5.4	1.4	0.7	1.7	21.4	80.44
3	4.9	0.1	0.7	1.3	16.2	84.69
4	5.7	2.3	0.8	1.8	19.6	78.94
5	6.1	0.1	1.2	2.5	28.3	74.74
6	5.2	0.6	0.7	1.8	21.4	81.17
7	4.09	0.5	0.6	1.5	15.9	84.92
8	5.9	1.8	0.9	1.8	19.2	78.92
9	4.8	3.7	0.5	0.8	16.1	83.35
10	6.4	1.04	0.8	1.7	13.4	81.65
Mean	5.54	0.93	0.78	1.47	15.6	82.50
